# The Importance of Spatial Visual Scene Parameters in Predicting Optimal Cone Sensitivities in Routinely Trichromatic Frugivorous Old-World Primates

**DOI:** 10.3389/fncom.2018.00015

**Published:** 2018-03-27

**Authors:** Tristan Matthews, Daniel Osorio, Andrea Cavallaro, Lars Chittka

**Affiliations:** ^1^Centre for Intelligent Sensing, Queen Mary University of London, London, United Kingdom; ^2^School of Life Sciences, University of Sussex, Brighton, United Kingdom; ^3^School of Biological and Chemical Sciences, Queen Mary University of London, London, United Kingdom; ^4^Institute for Advanced Study, Berlin, Germany

**Keywords:** colour vision, cones, parvocellular, photoreceptor cells, primate, red-green system, spatio-chromatic, spectral sensitivity

## Abstract

Computational models that predict the spectral sensitivities of primate cone photoreceptors have focussed only on the spectral, not spatial, dimensions. On the ecologically valid task of foraging for fruit, such models predict the M-cone (“green”) peak spectral sensitivity 10–20 nm further from the L-cone (“red”) sensitivity peak than it is in nature and assume their separation is limited by other visual constraints, such as the requirement of high-acuity spatial vision for closer M and L peak sensitivities. We explore the possibility that a spatio-chromatic analysis can better predict cone spectral tuning without appealing to other visual constraints. We build a computational model of the primate retina and simulate chromatic gratings of varying spatial frequencies using measured spectra. We then implement the case study of foveal processing in routinely trichromatic primates for the task of discriminating fruit and leaf spectra. We perform an exhaustive search for the configurations of M and L cone spectral sensitivities that optimally distinguish the colour patterns within these spectral images. Under such conditions, the model suggests that: (1) a long-wavelength limit is required to constrain the L cone spectral sensitivity to its natural position; (2) the optimal M cone peak spectral sensitivity occurs at ~525 nm, close to the observed position in nature (~535 nm); (3) spatial frequency has a small effect upon the spectral tuning of the cones; (4) a selective pressure toward less correlated M and L spectral sensitivities is provided by the need to reduce noise caused by the luminance variation that occurs in natural scenes.

## 1. Introduction

Young's (1802) trichromatic theory of colour vision recognises that the retina must compromise between spectral and spatial sampling of the optical image. The implications of the dual function for the retina remain to be fully explored, but there is much diversity amongst vertebrates in both photoreceptor spectral sensitivities and their spatial layout, which may depend both on retinal physiology and visual ecology (Osorio and Vorobyev, 2005). Primate trichromacy is of particular interest because of the uneven spectral distribution of the S, M, and L cones (at 440, 535, and 562 nm, respectively; Stockman and Sharpe, 2000; Surridge et al., 2003), the apparently random arrangement of L and M cones in varying ratios (Mollon and Bowmaker, 1992; Bowmaker et al., 2003), and the fact that both L and M cones contribute to the luminance mechanism.

Most accounts of primate trichromacy evaluate colour discrimination for tasks such as finding food without regard to the spatial layout of the cones (Mollon, 1989; Osorio and Vorobyev, 1996; Regan et al., 1998; Sumner and Mollon, 2000a; Dominy and Lucas, 2001; Lewis and Zhaoping, 2006; Melin et al., 2013). Generally, these studies predict the optimal value of $\lambda_{max}^{\text{M}}$ closer to $\lambda_{max}^{\text{L}}$ than to $\lambda_{max}^{\text{S}}$, but, nonetheless, at a wavelength 10–20 nm shorter than its actual value of 535 nm. To account for the disparity between the observed and predicted values of $\lambda_{max}^{\text{M}}$ it is sometimes proposed that the spectral separation of the L and M pigments is limited by the costs to spatial vision of having spectrally separated inputs to the luminance system (Mollon, 1989; Williams et al., 1991; Nagle and Osorio, 1993; Osorio et al., 1998; Rucker and Osorio, 2008). If there is indeed a trade-off between spatial and chromatic coding in natural images, one might speculate that the observed value of $\lambda_{max}^{\text{M}}$ corresponds to the optimal compromise to the problem recognised by Young (1802).

Psychophysical experiments comparing trichromats with dichromats that lack either M or L cones would support a trade-off of spatial and colour vision if the trichromats demonstrated inferior luminance vision due to the decorrelation of their M and L cone spectral sensitivities. Dichromats have been shown to have a foraging advantage under certain conditions, such as laboratory experiments (Jägle et al., 2006; Janáky et al., 2014), low-light (Caine et al., 2010), and discovering colour-camouflaged insects (Melin et al., 2007, 2010). However, single-cell recordings from the lateral geniculate nucleus of dichromatic and trichromatic marmosets suggest that the evolution of red-green colour vision has not come at a cost for spatial vision (Martin et al., 2011). Therefore, it appears that while there is a trade-off between spatial and chromatic vision, it is not pervasive across viewing scenarios, and its cause at a neural level has not yet been identified.

What is missing from previous models that predict the optimal cone spectral sensitivities is a consideration of spatio-chromatic signals that takes account of how the trichromatic eye encodes coloured patterns. Models of purely spectral coding (e.g., Osorio and Vorobyev, 1996), generally overlook the consequences of the spatial properties of the receptor array, and the fact that colour vision has to locate small objects, such as fruit amongst leaves, despite its relatively poor spatial acuity (Mullen, 1985; but see: Párraga et al., 2000, 2002). Our goal is to determine whether the optimal primate spectral sensitivities can be better predicted via a spatio-chromatic analysis than via a purely spectral analysis that does not consider spatial dimensions. We follow conventional understanding in assuming that the primate colour vision system has three main components (Mollon, 1989; Regan et al., 2001): the luminance system, which benefits from closer $\lambda_{max}^{\text{M}}$ and $\lambda_{max}^{\text{L}}$; the blue-yellow opponent system, which benefits from large separation of $\lambda_{max}^{\text{S}}$ from $\lambda_{max}^{\text{M}}$ and $\lambda_{max}^{\text{L}}$, and the red-green opponent system, which is a relatively recent evolutionary innovation unique to primates.

Primates include a diverse range of visual phenotypes, including dichromats, polymorphic trichromats and routine trichromats (for an extensive review, see Surridge et al., 2003). The model presented in this paper is intended to be a general model of primate retinal processing for spatio-chromatic spectral sensitivity prediction in primate/mammalian species. We use the case study of catarrhines foraging for fruit to guide model parameter settings. In addition to the peak sensitivities mentioned above, we make the following assumption: the catarrhine foveola is the key retinal region underlying red-green spatio-chromatic vision (Martin et al., 2011), so the greatest influence on spectral tuning can be captured by modelling only L and M cones because these are the majority, if not sole, photoreceptor class in the foveola (Ahnelt and Kolb, 2000), and only the single-cone centre midget retinal ganglion cells (Lennie et al., 1991). We refer to this model as a “parvocellular” model, though it does not include the P cells of the LGN.

We develop a computational spatio-chromatic model of the primate retina and implement the case study of the catarrhine foveola and foraging for fruits. We use the model to predict performance as a function of M and L cone spectral sensitivities under various conditions, then relate the model predictions to observations of photoreceptor spectral tuning. We test the model on databases of simulated spectral images, produced by placing recorded spectra of fruit and leaves into grating patterns. Given a physiologically realistic retina model which spatio-chromatically predicts the optimal M and L cone spectral sensitivities, we perform four sets of simulations to address the following questions: (i) How does varying the set of spectra used to model the visual environment affect the predicted M and L spectral sensitivities? (ii) Is a long-wavelength limit required to constrain the L cone spectral sensitivity to its observed position? (iii) Can better predictions of the optimal spectral sensitivities be achieved by a spatio-chromatic analysis than a purely spectral analysis? (iv) Does the variation in luminance that occurs across the spatial dimensions in natural scenes affect the optimal position of the M cone spectral sensitivity?

## 2. Model

We build a computational model of the retinal aspects of the primate parvocellular channel to determine the performance of all $\lambda_{max}^{\text{M}}$ and $\lambda_{max}^{\text{L}}$ combinations for the task of detecting patterns composed of fruit and leaf spectra. We use datasets of two-colour grating patterns containing fruit or leaf spectra at each pixel: the spatio-chromatic challenge is to discriminate regions of one class (material) from those of the other. To investigate the effects of spatial frequency on spectral sensitivity tuning, we run separate simulations on gratings at a range of spatial frequencies.

**Figure 1:**
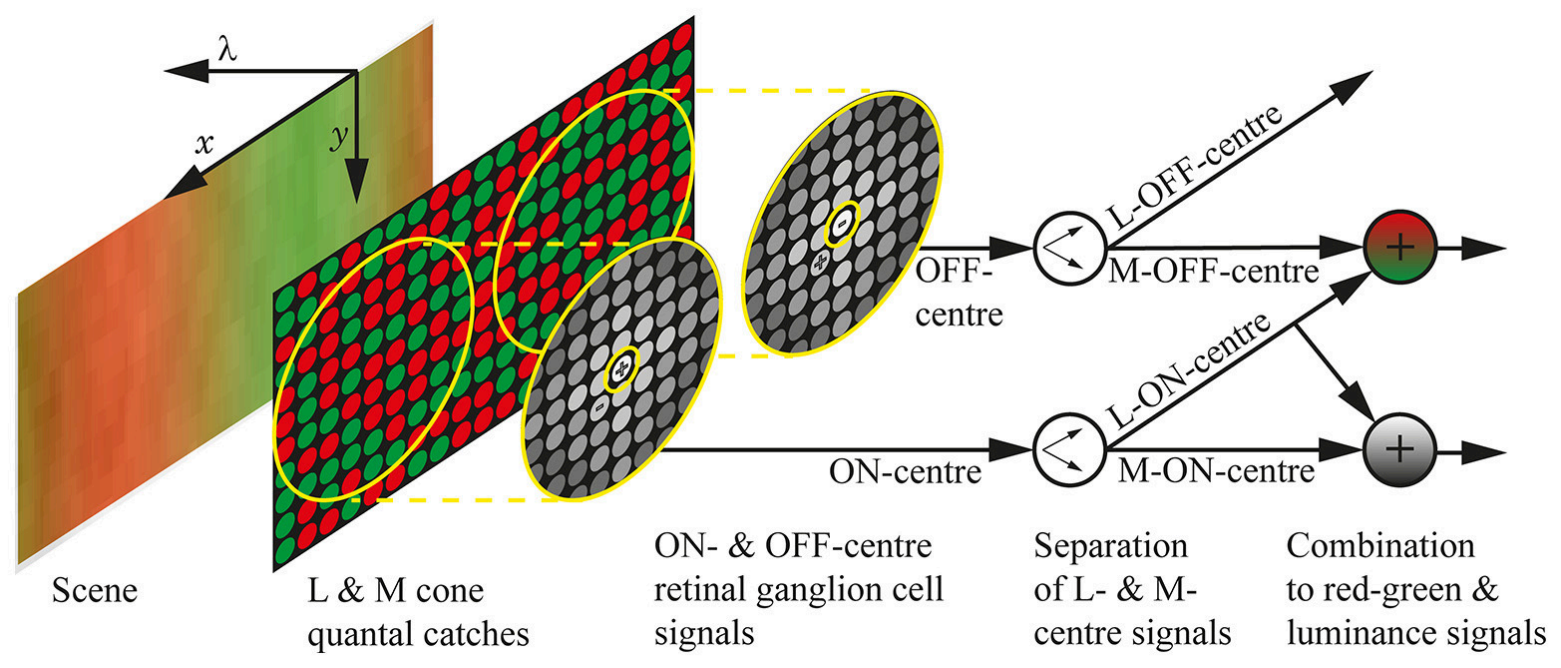
The neural systems included in the model. The visual scene is a section of a chromatic grating, with the spatial dimensions marked as *x* and *y*, and the spectral dimension marked as λ. The quantal catches of the M and L cone photoreceptors are illustrated as green and red circles, respectively, and the cones are randomly arranged in the photoreceptor mosaic. The retinal ganglion cell receptive fields are shown as yellow concentric circles, with the approximate weighting of the cones depicted as greyscale circles and the + and − indicating whether the sub-unit (centre or surround) is ON or OFF. The centres take input from only a single cone, while the surrounds take input from multiple cones and do not discriminate between M and L types; the size of the receptive fields is approximate for 3 standard deviations. We assume that the cortex can learn the centre identity of retinal ganglion cells (Wachtler et al., 2007), which we represent here as a separation of ON-centre signals into L-ON-centre and M-ON-centre signals, and OFF-centre signals into L-OFF-centre and M-OFF-centre signals. Both L-ON-centre and M-OFF-centre are excited when the L cones in their receptive fields are relatively more active than the M cones, so their combination (a matrix summation) gives the final red-green signal, which holds high values in red regions, low values in green regions, and gives complete coverage of the scene. Similarly, the luminance signal is formed by a combination of L-ON-centre and M-ON-centre signals. The design of this model was inspired by that presented in Stockman et al. (2014).

**Figure 2:**
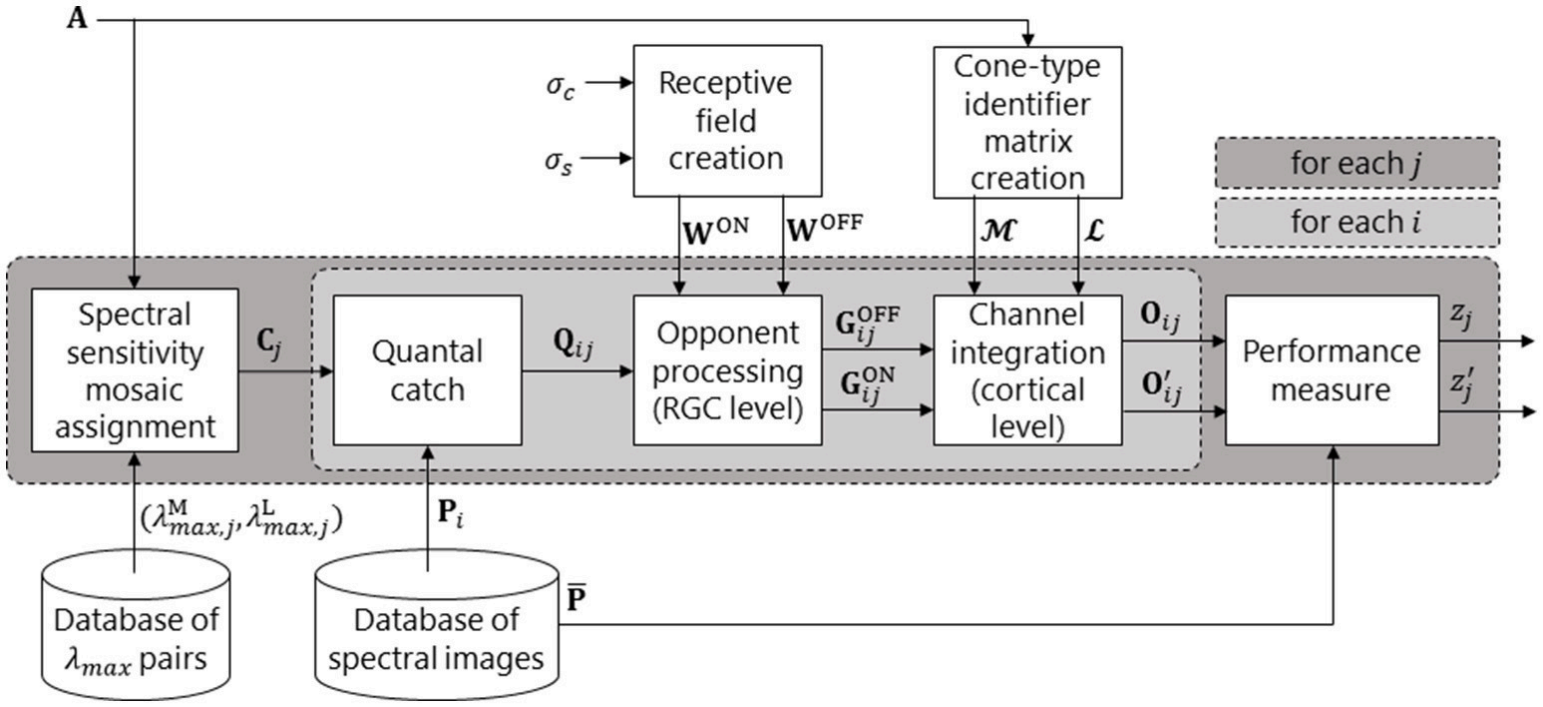
Pipeline of our retina model. The dark grey box represents a loop over all spectral sensitivity pairs. The light grey box represents a loop over all spectral images (and is the biological model, as depicted in Figure 1). For a given pair of peak sensitivities ($\lambda_{\text{max,j}}^{\text{M}}$, $\lambda_{\text{max,j}}^{\text{L}}$), an M and an L cone spectral sensitivity is created. These are then assigned to the M and L positions in mosaic **A** to produce spectral sensitivity mosaic **C**_*j*_. A given spectral image, **P**_*i*_, is then presented to the system and the quantal catch matrix, **Q**_*ij*_, is calculated. The retinal ganglion cell (RGC) layer performs colour opponent processing (via centre-surround opponent receptive fields) to create a matrix of OFF-centre RGC responses, ${\text{G}}_{ij}^{\text{OFF}}$, and a matrix of ON-centre RGC responses, ${\text{G}}_{ij}^{\text{ON}}$. In the Channel integration block, the ${\text{G}}_{ij}^{\text{ON}}$ and ${\text{G}}_{ij}^{\text{OFF}}$ matrices are split into ${\text{L}}_{ij}^{\text{ON}}$, ${\text{M}}_{ij}^{\text{ON}}$, ${\text{L}}_{ij}^{\text{OFF}}$, and ${\text{M}}_{ij}^{\text{OFF}}$ matrices via the cone-type identifier matrices, L and M. The red-green signal is then created as **O**_*ij*_ = ${\text{L}}_{ij}^{\text{ON}}+{\text{M}}_{ij}^{\text{OFF}}$ and the luminance signal as $O_{ij}^{\prime }$ = ${\text{L}}_{ij}^{\text{ON}}$ + ${\text{M}}_{ij}^{\text{ON}}$. This concludes the biological model. The remaining block produces a measure, *z*_*j*_, of performance of the *j*^*th*^ spectral sensitivity pair via comparison of **O**_*ij*_ or $O_{ij}^{\prime }$ with the reference pattern, $\bar{\text{P}}$.

Figure 1 presents the neural systems included in our model of parvocellular processing for the red-green and luminance systems. Figure 2 presents the full processing pipeline, with the neural systems inside the central light grey box. The model produces a performance score, *z*_*j*_, for each spectral sensitivity pair $\left(\lambda_{max,j}^{\text{M}},\lambda_{max,j}^{\text{L}}\right)$, by testing the ability of $\left(\lambda_{max,j}^{\text{M}},\lambda_{max,j}^{\text{L}}\right)$ to spatio-chromatically distinguish fruit and leaf regions across a database of spectral images. A spectral image is an image of size *N*_*x*_ × *N*_*y*_ × *N*_λ_, where *N*_*x*_ and *N*_*y*_ are the sizes of the horizontal and vertical spatial dimensions, and *N*_λ_ is the number of wavelength samples in the spectral dimension. Only two spectra—one fruit and one leaf—are used to build each spectral image.

### 2.1. Spectral sensitivity mosaic creation

We simulate a spatio-spectral photoreceptor mosaic, **C**, of size *N*_*x*_ × *N*_*y*_ × *N*_λ_ by assigning a cone spectral sensitivity of length *N*_λ_ in each location of an *N*_*x*_ × *N*_*y*_ spatial mosaic. Let $\left(\lambda_{max,j}^{\text{M}},\lambda_{max,j}^{\text{L}}\right)$ be the *j*th pair of peak sensitivity values drawn from a database of *N*_*s*_ pairs. Let *A* **=** (_*a*_*xy*_)*N*_*x*_ × *N*_*y*__ be a matrix describing the spatial arrangement of M and L cones, where the elements are cone type labels: *a*_*xy*_ ∈ {*M, L*}. Let $\psi \left(\lambda ,\lambda_{max,j}^{a_{xy}}\right)$ be a function that returns the value at wavelength λ of a spectral sensitivity with peak sensitivity at $\lambda_{max,j}^{a_{xy}}$. To build the spatio-spectral mosaic for the *j*th spectral sensitivity pair, we extend the formulation in Sumner and Mollon (2000b) to include spatial dimensions as:

(1) $$C_{j}\left(x,y,\lambda \right)=10^{-\Delta \left(\lambda \right)}\psi \left(\lambda ,\lambda_{max,j}^{a_{xy}}\right)\;,$$

where *x* and *y* are the cone (pixel) coordinates; λ is wavelength sampling positions drawn from the *N*_λ_-length vector of all wavelength sampling positions, Λ; and Δ(λ) gives the combined wavelength-dependent optical densities of the lens and macular pigment at wavelength λ. Thus, Equation (1) gives the spectral sensitivity at wavelength λ of a cone of type *a*_*xy*_ (either M or L), adjusted to account for the ocular media.

### 2.2. Quantal catch

To simulate viewing of the scene, we present the system with a spectral image, **P**_*i*_, of the same spatial and spectral dimensions as **C**_*j*_ (*N*_*x*_ × *N*_*y*_ × *N*_λ_) and follow previous works (Kelber et al., 2003) by calculating quantal catch at each spatial location to produce an *N*_*x*_ × *N*_*y*_ quantal catch matrix, **Q**_*ij*_, where:

(2) $$Q_{ij}\left(x,y\right)=\sum_{\lambda \in \Lambda }P_{i}\left(x,y,\lambda \right)C_{j}\left(x,y,\lambda \right).$$

It is assumed that the illuminant is already included with the spectral image. Notice that Equation (2) does not discriminate between cone types, so matrix **Q**_*ij*_ contains the quantal catches of both M and L cones.

### 2.3. Receptive field creation

We model the opponent processing of retinal ganglion cells (RGCs) as Difference of Gaussians (Soodak, 1986). Let **W**^ON^ be an ON-centre OFF-surround receptive field and **W**^OFF^ be an OFF-centre ON-surround receptive field. Each of these is formed as a two-dimensional circularly symmetrical Difference of Gaussians. Because of the symmetry, we define a 2D Gaussian, N(μ, σ), using a scalar mean, μ, and scalar standard deviation, σ, as:

(3) $$N\left(\mu ,\;\sigma \right)=\exp\left(-\left(\frac{{\left(x-\mu \right)}^{2}+{\left(y-\mu \right)}^{2}}{2\sigma^{2}}\right)\right).$$

The two receptive fields can then be defined as sums of zero-mean Gaussians with different standard deviations, as:

(4) $$\begin{array}{l} W^{\text{ON}}=N\left(0,\sigma_{c}\right)-\omega N\left(0,\sigma_{s}\right),\\ W^{\text{OFF}}=-W^{\text{ON}}, \end{array}$$

where σ_*c*_ and σ_*s*_ are the scalar standard deviations of the centre and surround Gaussians, respectively, and ω allows the sensitivity of the surround receptive field to be adjusted with respect to that of the centre.

Note that the receptive field does not discriminate between cone types, so this is a non-selective receptive field model. In order to achieve colour opponency, the centre must have high cone purity (draw input predominantly from one cone type). In the present work, we only model the single-cone centres of RGCs in the foveola, so cone purity of centres is 100%.

### 2.4. Opponent processing

Next, we model the opponent processing of the ON-centre and OFF-centre RGCs. It is likely that midget RGCs cannot discriminate between L and M cones (Paulus and Kroger-Paulus, 1983; Benson et al., 2014), so let all ON-centre RGC responses be held in a *N*_*x*_ × *N*_*y*_ matrix, ${\text{G}}_{\text{ij}}^{\text{ON}}$. Moreover, let ${\text{G}}_{\text{ij}}^{\text{OFF}}$ be an equivalent matrix of OFF-centre RGC responses. These are computed as:

(5) $$\begin{array}{l} G_{ij}^{\text{ON}}\;=\;Q_{ij}*W^{\text{ON}},\\ G_{ij}^{\text{OFF}}\;=\;Q_{ij}*W^{\text{OFF}}, \end{array}$$

where ^*^ represents the convolution operation. Notice that both cone types (M and L) in **Q**_*ij*_ are involved in the convolution; i.e., this is a model of non-selective RGC centres and surrounds, so colour opponency is achieved by the fact that the centre contains only a single cone—the spectral sensitivity of the single-cone centre is exactly the same as that of its single cone, while the spectral sensitivity of the surround is a mixture of the spectral sensitivities of M and L cones.

The convolution operation in Equation (5) produces *N*_*w*_ − 1 pixels that incur border effects in ${\text{G}}_{\text{ij}}^{\text{ON}}\;$and ${\text{G}}_{\text{ij}}^{\text{OFF}}$, where *N*_*w*_ is the length of each dimension of the window containing the receptive field. These are artefacts, they are removed prior to measuring performance.

### 2.5. Cone-type identifier matrix creation

The quantal catches of both cone types are contained within the same matrix, **Q**_*ij*_, but subsequent steps treat M and L cones differently. From the given photoreceptor mosaic, we create two logical matrices, L and M, which act as pointers to cones of their respective type in **Q**_*ij*_, as:

(6) $$\begin{array}{l} \;L={\left(l_{xy}\right)}_{N_{x}\times N_{y}},\\ \;l_{xy}=\{\begin{array}{l} 0\\ if\;a_{xy}=\text{M }\\ 1\\ if\;a_{xy}=\text{L } \end{array},\\ M={\left(m_{xy}\right)}_{N_{x}\times N_{y}},\;\\ m_{xy}=1-l_{xy}. \end{array}$$

### 2.6. Channel integration

We now have two matrices of RGC responses: ${\text{G}}_{\text{ij}}^{\text{ON}}$ contains both L ON-centre and M ON-centre (we will refer to these as ${\text{L}}_{\text{ij}}^{\text{ON}}$ and ${\text{M}}_{\text{ij}}^{\text{ON}}$) responses, and ${\text{G}}_{\text{ij}}^{\text{OFF}}$ contains both L OFF-centre and M OFF-centre (${\text{L}}_{\text{ij}}^{\text{OFF}}$ and ${\text{M}}_{\text{ij}}^{\text{OFF}}$) responses. To build a red-green signal, we require: (i) only the RGCs that are excited by “reddish” stimuli; (ii) RGCs with both M and L centre so as to achieve full coverage of the scene. These two conditions are met by ${\text{L}}_{\text{ij}}^{\text{ON}}$ and ${\text{M}}_{\text{ij}}^{\text{OFF}}$. Assuming the cortex can learn RGC centre identities from the statistics of their activations (Wachtler et al., 2007), then $L_{ij}^{\text{ON}}=LG_{ij}^{\text{ON}}$ and $M_{ij}^{\text{OFF}}=MG_{ij}^{\text{OFF}}$. So, the red-green matrix of outputs to the cortex, **O**_*ij*_, is calculated as:

(7) $$O_{ij}=LG_{ij}^{\text{ON}}+MG_{ij}^{\text{OFF}}.$$

Similarly, the luminance signal, $O_{j}^{\prime }$, is a combination of L and M centre RGC signals for full scene coverage, but only those with ON-centre receptive fields (which is equivalent to ${\text{G}}_{\text{ij}}^{\text{ON}}$). This is calculated as:

(8) $$O_{ij}^{\prime }=LG_{ij}^{\text{ON}}+MG_{ij}^{\text{ON}}=G_{ij}^{\text{ON}}.$$

Note that the matrices **O**_*ij*_ and $O_{ij}^{\prime }$ are *inputs* to the cortex; therefore, no attempt has been made to model the spatial integration that is thought to occur in the cortex, but for which the mechanism has not yet been revealed (Solomon and Lennie, 2007).

## 3. Settings for the parvocellular channel

This section explains the parameter settings of the simulations. We first specify the parameter settings of the biological model, including the photoreceptor mosaic, spectral sensitivities and receptive fields. Following this, we describe the creation of the database of spectral images, and finish by describing the performance measure.

### 3.1. Animal model

Ideally, we would use the parameter settings (e.g., focal length, transmission of optical media) of trichromatic primates at the time when they diverged from dichromats. However, such information is not available. Unless otherwise stated, we use humans as models since the data is more comprehensive than for other species, and, where comparisons have been made, values tend to be similar across primate species (Cooper and Robson, 1969; Snodderly et al., 1984; Tovée et al., 1992).

### 3.2. The photoreceptor mosaic

For computational convenience, we model the photoreceptor mosaic as a square grid (Benson et al., 2014). This is a simplification of the hexagonal grid found in the primate eye (Hofer et al., 2005) that eases the computation and description, but does not affect our results because within one simulation we compare all spectral sensitivity pairs on exactly the same mosaic. For convenience and efficiency, we model one cone as equal in size to one pixel. In the human fovea, the minimal centre-to-centre spacing of cones is $\frac{1}{120}$ degrees visual arc, or 2.5 μm (Wandell, 1995), so we define one pixel as $\frac{1}{120}\times \frac{1}{120}$ degrees visual arc.

To demonstrate the advantage of spatio-chromatic modelling to estimate optimal spectral sensitivity tuning, we focus upon the spectral tuning of the cones that underlie the red-green system. For this reason, we only model the central region of the primate foveola, which contains no S cones. In humans, the S-cone-free region is around 20/60 degrees across (100 μm, or 40 cones) (Bumsted and Hendrickson, 1999), though it is smaller in macaques, at around 9/60 degrees across (45 μm, or 18 cones) (de Monasterio et al., 1985). However, varying the size of the mosaic does not affect any calculations, so we do not strictly limit ourselves to the size found in nature. Instead we choose the horizontal and vertical photoreceptor mosaic dimensions, *N*_*x*_ and *N*_*y*_, to match the size of the spectral images.

For the L:M ratio of the photoreceptor mosaic, we use 1:1. This ratio has been observed as average in most non-human primates (Mollon and Bowmaker, 1992), so we assume the earliest trichromatic ancestor was similar. Though large variation has been observed in this ratio (Deeb et al., 2000), we do not include this in our simulation because it is not clear whether this variation existed in the earliest primate that evolved trichromacy and it is also not yet clear what mechanisms assure similar colour vision performance across different L:M ratios (Brainard et al., 2000).

To assign cone identities to the mosaic, let **A** = (_*a*_*xy*_)*N*_*x*_ × *N*_*y*__ be the photoreceptor mosaic, and *a*_*xy*_ ∈ {*M, L*} be the cone identity at pixel (*x, y*). In the primate retina, L and M cones have a locally random arrangement (Mollon and Bowmaker, 1992). Thus, we randomly assign each *a*_*xy*_ with a 0.5 chance to be M, otherwise it is L.

### 3.3. Spectral sensitivities

For the combined wavelength-dependent optical densities of the lens and macular pigment, Δ, we follow Sumner and Mollon (2000a) in using those of humans, as given in Wyszecki and Stiles (1982). For the function which generates the spectral sensitivities from the peak sensitivities, ψ(·, ·), we use the peak wavelength-dependent nomogram described in Stockman and Sharpe (2000) and the resulting absorbance curves are converted to absorptance curves.

### 3.4. Receptive fields

The size of the receptive fields is chosen to match that in the primate fovea in having a single cell centre (Calkins and Sterling, 1996), which is achieved by σ_*c*_ = 0.25 cones (95% of the volume of a Gaussian falls within 2 standard deviations either side of the mean, and 4σ_*c*_ = 1). The size of the surround is determined from the centre-to-surround ratio as: σ_*c*_/σ_*s*_ = 0.15 (Croner and Kaplan, 1995), which yields σ_*s*_ = 1.66 cones (95% of the Gaussian is covered by a diameter of 4σ_*s*_ = 6.67 cones). *N*_*w*_, the length of each dimension of the window that contains the receptive field, is set to 9 cones. This accommodates slightly less than three standard deviations each side of the mean (6σ_*s*_ = 9.96), but allows us to keep a single cone in the centre of the window. For our fixed receptive field size, we verified that this window size did not influence results by running a comparison test with *N*_*w*_ = 11 cones and observed no difference in the results. It has been shown that the relative sensitivities of centre and surround are highly variable from cell to cell, but that the sensitivity of the surround is, on average, 55% weaker than that of the centre (Croner and Kaplan, 1995). We use this average value by setting ω = 0.55.

### 3.5. Spectral image database

**Figure 3:**
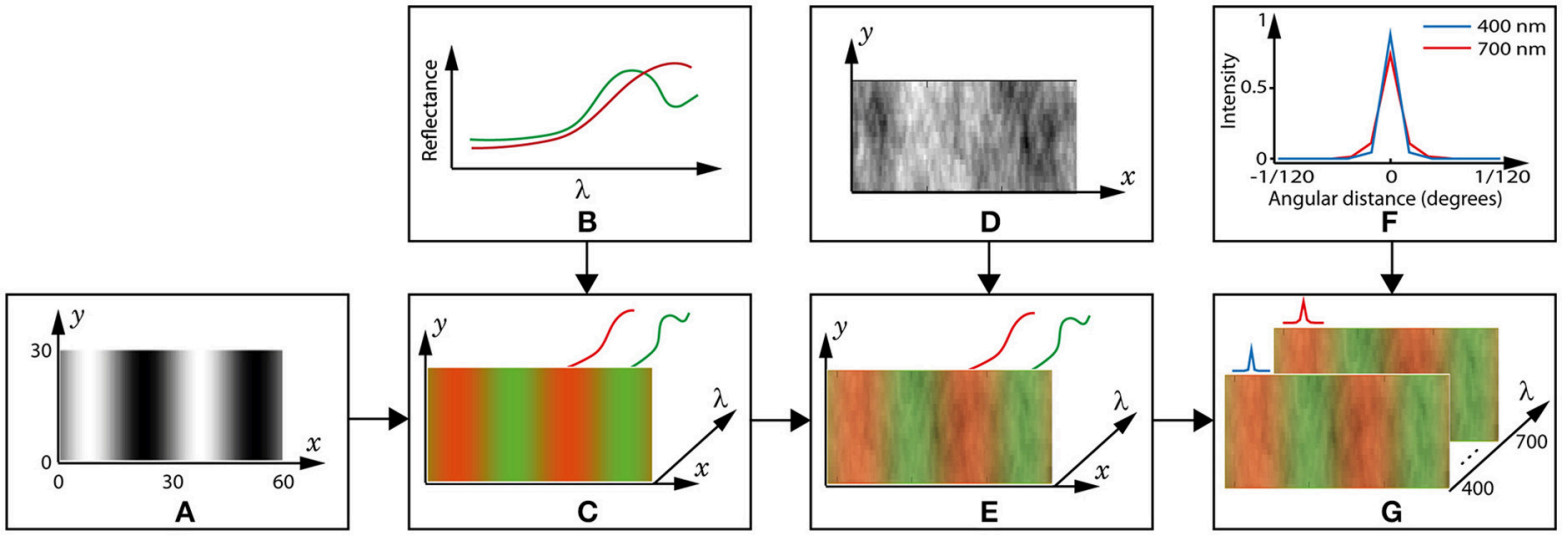
Creation of a spectral image. **(A)** The reference pattern, $\bar{\text{P}}$, is a 2D matrix in which the black and white pixels represent leaf and fruit positions, respectively. The axis dimensions are in pixels, and 1 pixel = 1 cone = 1/120 degrees visual arc. This example grating is 4 cycles per degree (15 pixels per bar). **(B)** Example spectra of a fruit (red) and leaf (green). **(C)** A 3D matrix is created by placing a leaf spectrum in the wavelength (**λ**) dimension in each black pixel in the reference pattern, a fruit spectrum in each white pixel and a weighted mixture at the grey pixels. The colours are for convenience and to match those used for the spectra in **(B)**. Though not shown, the illuminant is applied to each spectrum at this step. **(D)** The coefficients used to model luminance variation are contained in a 2D matrix of the same dimensions as $\bar{\text{P}}$. **(E)** luminance variation is included in the 3D image by multiplying all values in the **λ** dimension by the coefficient in the respective (x,y) pixel position. **(F)** The point-spread function (PSF) of the lens is different at each wavelength. The blue and red lines show cross-sections of the PSF at 400 and 700 nm, respectively, and for all other wavelengths the PSF is between these extremes. **(G)** The spectral image, **P**_*i*_, is completed by convolving each wavelength layer with its respective PSF.

We use grating patterns rather than images of natural scenes because this allows us to vary spatial frequency in a well-defined manner. To emulate the sinusoidal gratings commonly used in psychophysical studies (Mullen, 1985; Brainard et al., 2008; Lee et al., 2012), we create two-colour grating patterns containing ecologically valid reflectance spectra in each pixel (spectral images). Each spectral sensitivity pair's performance is measured as its ability to maximise the distinctiveness of the spatial pattern; i.e., the sinusoidal grating. The highest spatial frequency used is 4 cycles per degree, as it has been shown that significant chromatic aberration affects the retinal image at spatial frequencies above 4 cycles per degree (Flitcroft, 1989). The exact spatial frequencies, in cycles per degree, are {4, 2, 1, 0.5}, which equates to {30, 60, 110, 222} cones (1 cone = 1 pixel) per cycle, or $\left\{\frac{15}{60},\frac{30}{60},\frac{55}{60},\frac{111}{60}\right\}$ degrees visual arc, respectively. The creation of a spectral image is demonstrated in Figure 3, and other spectral images vary in the spatial frequency of the grating (A), the fruit spectrum (B), and the set of luminance coefficients (D).

All spectral images in one simulation use the same spatial pattern: $\bar{P}\;=\;{\left({\bar{p}}_{xy}\right)}_{N_{x}\times N_{y}},{\bar{p}}_{xy}\in [0,\;1]$, which is also used as the reference pattern with which the model outputs are compared. For each simulation, we create the database of spectral images, ${\text{P}}_{i}\in {\mathbb{R}}^{N_{x}\times N_{y}\times N_{\lambda }},\;i=1,2,\ldots ,N_{f}$. Let ${\text{s}}_{i}\in {\mathbb{R}}^{N_{\lambda }}$ be the *i*th target spectrum in the database of *N*_*f*_ target spectra, and let ${\text{s}}_{b}\in {\mathbb{R}}^{N_{\lambda }}$ be the mean of all background leaf spectra. For each pixel position (*x, y*) in **P**_*i*_, the spectrum is composed from the target and background incident spectra, **s**_*i*_ and **s**_*b*_, as ${\;\bar{p}}_{xy}{\text{s}}_{i}+\left(1-\;{\bar{p}}_{xy}\right){\text{s}}_{b}$.

The spectra are taken from the Cambridge database of natural spectra (Regan et al., 1998; Sumner and Mollon, 2000b), which contains a variety of spectra recorded from fruits eaten by several primate species, and mature leaves from the same environments. The spectra are provided at 4 nm sampling intervals, and we truncate them to the primate visual range of 400–700 nm, so *N*_λ_ = 76 and Λ = {400, 404, 408, …, 700}. The number of images in the database matches the number of spectra in the Cambridge database: *N*_*f*_ = 1139 fruits. Note that each spectral image, **P**_*i*_, is created from only two spectra: one target and one background, where the background spectrum is always the mean of all (409) mature leaves, as has been done in previous work (Chittka and Menzel, 1992; Osorio and Vorobyev, 1996). We also tested using randomly selected background leaf spectra in each pixel rather than the mean, but this had no effect on the results. For one set of simulations, we use spectra of Munsell chips (Parkkinen et al., 1989). In this case, there is no constant background spectrum for all images. Instead, for each image, we draw two spectra from the dataset randomly and without replacement.

Prior to building the images, the illuminant was added to all spectra as: **s** = **i** ⊙ **s**′, where **s**′ is the original spectrum, $\text{i}\in {\mathbb{R}}^{N_{\lambda }}$ is the illuminant, and ⊙ is the element-wise multiplication operator. We tested using three different illuminants: the D65 standard illuminant and two for forest areas in French Guiana and Uganda. We found that all three illuminants resulted in the same set of optimal spectral sensitivities, and so have selected to present only the results produced using the Ugandan forest illuminant from the Cambridge dataset (Regan et al., 1998; Sumner and Mollon, 2000b).

In natural scenes, there is far more variance in luminance compared to chromaticity (Ruderman et al., 1998; Wachtler et al., 2001), mainly due to the effects of shadows. Such effects are characterised as multiplicative factors on the reflected spectrum (Rubin and Richards, 1982). Though natural scenes may also contain some additive effects due to reflectance, we assume that these would be small enough to be negligible, as the specularities they cause would only occur when the surface was at a certain angle relative to the eye, and the animal could move its head to change this. Therefore, we do not include any additive reflectance effects. We included luminance variation by creating greyscale images which capture the 1/*f* power spectrum typically observed in natural images (Field, 1987; Billock, 2000; Millane et al., 2003), where *f* is the spatial frequency, and then using the pixel values as coefficients for the spectra in our images (1/*f* noise is also known as Pink noise). We used a set of 49 natural leafy scenes gathered from an internet search with the constraints that the images must contain only leaves, branches and sky, with branches and sky being minimal. These images were all converted to greyscale. We then performed a Fast Fourier Transform (FFT) on each image and used the median in each pixel value to create average magnitude and phase images. We verified that the resulting power spectrum was a close fit with the 1/*f* slope, then these median values were transformed back into the spatial domain via inverse FFT. An example luminance coefficient image is shown in Figure 3D.

The wavelength-dependent refractive effect of the lens is, strictly, a part of the biological model rather than the image model. However, as this is constant across all images and regardless of the cone spectral sensitivities, we include it in the spectral images. To account for the refractive effect of the lens, we apply a pixelwise point-spread function to all spectral images by convolving each wavelength layer of the spectral images with a wavelength appropriate Airy disk. The Airy disk is created using the data available for humans: lens refractive index = 1.406 (Garner et al., 1998), focal length = 21.3 mm (Van Norren and Tiemeijer, 1986), pupil diameter = 5 mm (Liang and Williams, 1997), and for each wavelength λ ∈ Λ. The line spread functions for the shortest and longest wavelengths we use, λ = 400 nm and λ = 700 nm, are shown in Figure 3F.

Vertical image size is *N*_*y*_ = 30 cones (pixels). Horizontal image size, *N*_*x*_, is dependent on spatial frequency, such that each image contains two cycles of the sinusoidal grating. Specifically, this gives *N*_*x*_ ∈ {60, 120, 220, 444} cones (pixels).

### 3.6. Performance measure

We measure the ability of each peak sensitivity pair $\left(\lambda_{max,j}^{\text{M}},\lambda_{max,j}^{\text{L}}\right)$, to facilitate the discrimination of chromatic gratings. We make the assumption that the cortical cells receiving input from the parvocellular channel attempt to estimate the input image (Manning and Brainard, 2010). The *j*th peak sensitivity pair's estimate of the *i*th image is stored in matrices of outputs to the cortex: **O**_*ij*_ for the red-green signal and $O_{ij}^{\prime }$ for the luminance signal. Moreover, as we simulate the spectral images, the reference pattern, $\bar{\text{P}}$, is known, so the performance is measured as the similarity between the reference pattern and the matrices of outputs to the cortex. Here, we will describe how the performance measure is calculated for the red-green signal by comparison of **O**_*ij*_ and $\bar{\text{P}}$. Performance of the luminance signal is measured in the same manner—simply replace **O**_*ij*_ with $O_{ij}^{\prime }$ and *z*_*j*_ with $z_{j}^{\prime }$.

After finding **O**_*ij*_ for all *i* = 1, 2, …, *N*_*f*_ images, we compare them all with the reference pattern, $\bar{\text{P}}$, and produce a single measure of the performance, *z*_*j*_, of spectral sensitivity pair *j*. Values in $\bar{\text{P}}$ vary from 1 in target locations to 0 in background locations, while **O**_*ij*_ contains positive values in cells which are redder than their local neighbourhood (determined by the size of the receptive field surround) and negative values in cells which are greener than their local neighbourhood. To ensure **O**_*ij*_ and $\bar{\text{P}}$ are in the same range, we normalise **O**_*ij*_ to [0, 1] prior to the comparison. As all spectral images in one simulation have the same spatial pattern, $\bar{\text{P}}$ is the same for all **P**_*i*_. To measure the similarity between a single **O**_*ij*_ and $\bar{\text{P}}$, we use the Peak Signal-to-Noise Ratio, which is commonly used in image processing for comparing a reference and filtered image (Drew and Bergner, 2007). Its calculation is based upon Mean Square Error, but taking into account the maximal possible signal power. As such, this performance is maximal for the spectral sensitivity pair that causes the greatest mean distance in red-green space between all fruits and the background leaves. The performance measure, *z*_*j*_, is calculated as:

(9) $$z_{j}\;=\;\sum_{i\;=\;1}^{N_{f}}\frac{PSNR\left(O_{ij},\bar{P}\right)}{N_{f}},$$

where *PSNR*(·, ·) is the is the Peak-Signal-to-Noise Ratio of the two arguments, defined as:

(10) $$PSNR\left(O_{ij},\bar{P}\right)=10{\text{ log}}_{10}\frac{peakval^{2}\;}{MSE\left(O_{ij},\bar{P}\right)},$$

where $MSE\left({\text{O}}_{ij},\bar{\text{P}}\right)$ is the Mean Square Error of its two arguments. In Equation (10), *peakval* = 1 is the peak value that the signal can take, and the logarithm converts the result to decibels. The spatial performance is implicit because the red-green value at a given location in **O**_*ij*_ is a function of the activations of all cones in a local neighbourhood (determined by the size of the RGC receptive field). **O**_*ij*_ will contain noise which arises because: (i) the receptive field surrounds synapse both M and L cones (they are non-selective), and the ratio of L:M cones differs from surround to surround due to the random mosaic arrangement; (ii) the luminance variation can cause the activations of the cones stimulated by the spectra of the same material to be less correlated than cones stimulated by the spectra of different materials, particularly for very similar spectra. Noise of the first type should be minimised by smaller $\lambda_{max}^{\text{L}}-\lambda_{max}^{\text{M}}$, while noise of the second type will favour some separation, with the amount being dependent upon the nature of the spectra. The best performing spectral sensitivity pair is the one that maximises the chromatic signal described by $\bar{\text{P}}$ while minimising noise.

## 4. Results

We address four questions: (i) How does varying the set of spectra used to model the visual environment affect the predicted M and L spectral sensitivities? (ii) Is a long-wavelength limit required to constrain the L cone spectral sensitivity to its observed position? (iii) Can better predictions of the optimal spectral sensitivities be achieved by a spatio-chromatic analysis than a purely spectral analysis? (iv) Does the variation in luminance that occurs across the spatial dimensions in natural scenes affect the optimal position of the M cone spectral sensitivity?

The search parameters for the four sets of simulations are given in Table 1 and described in detail below. Random photoreceptor mosaics were used in all simulations. As this causes some variation in the resulting optimal peak sensitivities, all simulations were repeated multiple times with differently seeded mosaics. One “set” of simulations includes all simulations for the different spatial frequencies of 4, 2, 1 and 0.5 cycles per degree. Our primary focus is the red-green system, but in the Optimal $\lambda_{max}^{\text{M}}$ simulations, we also provide results for the luminance system to illustrate the relative superiority of the red-green system on the fruit foraging task.

**Table 1:** Search parameters for the four sets of simulations.

	$\lambda_{max}^{\text{M}}$ **(nm)**	$\lambda_{max}^{\text{L}}$ **(nm)**	**Inc. (nm)**	**Dataset**	**Lum. var**.	**Reps**.
Varied spectra simulations	490–560	490–560	10	Munsell	Yes	50
Long-wavelength limit simulations	490–598	490–598	4	Camb.	Yes	50
Optimal $\lambda_{max}^{\text{M}}$ simulations	515–535	562	1	Camb.	Yes	100
luminance variation simulations	440–562	562	10	Camb.	No	100

*Inc., increment; Lum. var., luminance variation; Reps., repetitions; Camb., Cambridge database of natural spectra (Regan et al., 1998; Sumner and Mollon, 2000b)*.

### 4.1. Varied spectra simulations

We aim to determine whether a spatio-chromatic parvocellular channel model will predict highly overlapping M and L spectral sensitivities even if a spectrally rich model of the environment is used. This is a control to ensure that the spatio-chromatic model is not biased toward predicting spectral sensitivities similar to those found in primates. We use a database of spectra of Munsell chips (Parkkinen et al., 1989) to create the spectral images, vary both spectral sensitivities over the range: $\lambda_{max,j}^{\text{M}},\lambda_{max,j}^{\text{L}}\in \left\{490,\;500,\;510,\;\ldots ,\;550,\;560\right\}$, and apply the constraint $\lambda_{max,j}^{\text{L}}\geq \lambda_{max,j}^{\text{M}}$. Each simulation is repeated 50 times on differently seeded random mosaics.

**Figure 4:**
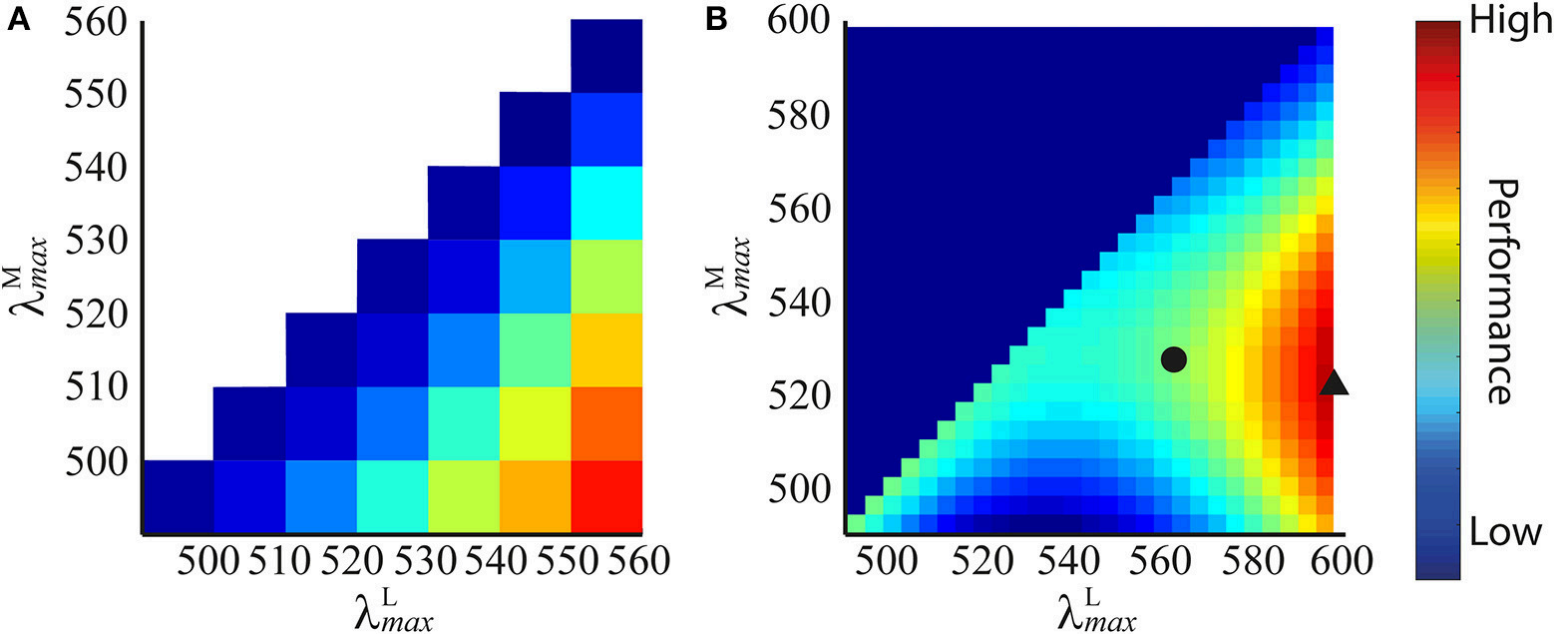
Performance plots for all pairs of peak sensitivities using spectral images created from spectra of **(A)** Munsell chips of wide variety of colours, and **(B)** fruit and leaves. The performance of each pair of peak sensitivities is represented by a colour: blues are low performance, reds are high performance (performance is *z* score (Equation 9) normalised to [0,1] for display purposes). On each dataset, the plots at all spatial frequencies were highly similar, so only those for 4 cycles per degree gratings are shown. In **(B)**, we highlight the absolute optimal peak sensitivity pair (black triangle) and the long wavelength limited ($\lambda_{max}^{\text{L}}\leq$ 562 nm) optimal peak sensitivity pair (black circle).

The results show that discrimination performance increases the more separated $\lambda_{max}^{\text{M}}\;$and $\lambda_{max}^{\text{L}}$ become, as shown in the performance plot in Figure 4A. Accordingly, the optimal peak spectral sensitivities are: $\lambda_{max}^{\text{M}}=$ 490 nm and $\lambda_{max}^{\text{L}}=$ 560 nm. Performance plots at all spatial frequencies were qualitatively similar, so only the result for 4 cycles per degree gratings is shown.

### 4.2. Long-wavelength limit simulations

We aim to determine whether a spatio-chromatic model requires a long-wavelength limit of $\lambda_{max}^{\text{L}}\leq$ 562 nm to constrain the L cone spectral sensitivity to its observed position, rather than a longer wavelength. We assume the task is foraging for fruit and perform an exhaustive search where both peak sensitivities, $\lambda_{max}^{\text{M}}$ and $\lambda_{max}^{\text{L}}$, vary. We apply the constraint that $\lambda_{max}^{\text{L}}\geq \lambda_{max}^{\text{M}}$. The minimum and maximum of 490 and 598 nm cover the range over which mammal LWS opsins exist (Jacobs, 2009), with an additional ~40 nm in the longwave direction to investigate whether the optimal L cone peak would lie at a longer wavelength than found in nature if no biological constraints applied, i.e., $\lambda_{max,j}^{\text{M}},\lambda_{max,j}^{\text{L}}\in \left\{490,\;494,\;498,\;\ldots ,\;594,\;598\right\}$. Each simulation is repeated 50 times on differently seeded random mosaics.

The results are presented in Figure 4B. The performance plots at all spatial frequencies were qualitatively similar, so only the result for 4 cycles per degree gratings is shown. On all simulations, $\lambda_{max}^{\text{L}}$ occupies the longest wavelength possible, and for any given $\lambda_{max}^{\text{M}}$, performance is always better if $\lambda_{max}^{\text{L}}>$ 562 nm. When both λ_*max*_ are at relatively short wavelengths (i.e., when the mean of the two is shorter than ~540 nm), discrimination performance is lower than the nearest configuration for which $\lambda_{max}^{\text{M}}=\lambda_{max}^{\text{L}}$ (at which point this ceases to be a red-green signal and becomes a luminance signal). The absolute optimum configuration occurs at $\lambda_{max}^{\text{L}}=$ 598 nm and $\lambda_{max}^{\text{M}}=$ 521.6 nm, whereas when the long-wavelength limit is applied, the optimum $\lambda_{max}^{\text{M}}$ is 525.8 nm. The extreme optimal $\lambda_{max}^{\text{M}}$ on individual runs were 518 and 526 nm—this variation is due to the random “seeding” of the photoreceptor mosaic between simulation repetitions and, to a lesser degree, of the luminance coefficients between images. These results guided us to limit the search range for subsequent simulations sets to 515 $\leq \lambda_{max}^{\text{M}}\leq$ 535 nm in 1 nm increments and $\lambda_{max}^{\text{L}}=$ 562 nm (discrimination performance is always better for $\lambda_{max}^{\text{L}}$ at longer wavelengths, so this is equivalent to $\lambda_{max}^{\text{L}}\leq$ 562 nm).

### 4.3. Optimal $\lambda_{max}^{\text{M}}$ simulations

We aim to determine whether the optimal position of the M cone spectral sensitivity is more accurately predicted by a spatio-chromatic analysis than by a purely spectral analysis in which spatial dimensions are not considered. We assume the ecological task of foraging for fruit, as this facilitates comparison with the purely spectral analyses of previous works (Osorio and Vorobyev, 1996; Regan et al., 1998; Sumner and Mollon, 2000a; Lewis and Zhaoping, 2006). We use the results of the long-wavelength limit simulations to limit our search space, thereby facilitating a higher-resolution (smaller increment between λ_*max*_ positions) search. As $\lambda_{max}^{\text{L}}$ always occupies the longest wavelength available, fixing $\lambda_{max}^{\text{L}}=$ 562 nm is equivalent to $\lambda_{max}^{\text{L}}\leq$ 562 nm. The search interval is reduced to 1 nm, and we increase the number of repetitions with differently seeded mosaics to 100. As our model produces both red-green and luminance signals that are sent to the cortex, we also demonstrate the spatio-chromatic advantage of the red-green system over the luminance system for the task of fruits foraging.

**Figure 5:**
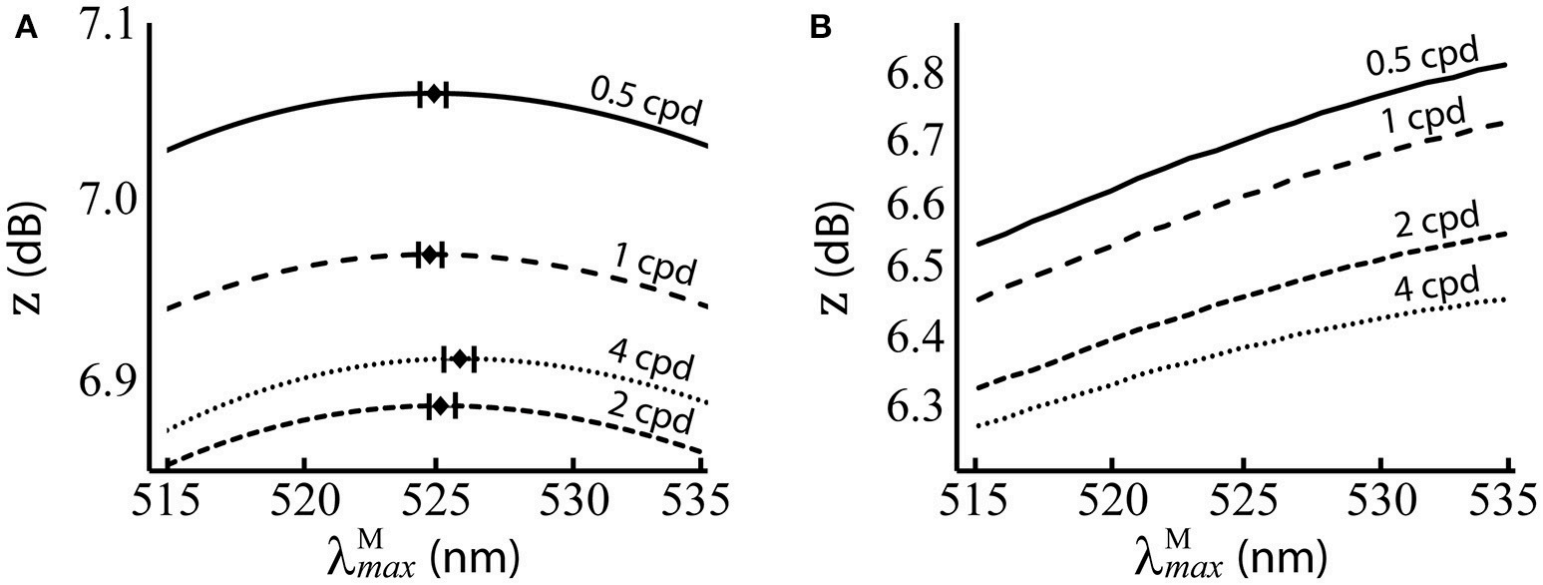
Performance of $\lambda_{\text{max}}^{\text{M}}$ at different spatial frequencies, when spectral images contain natural luminance variation. **(A)** Performance of the red-green system. Mean optimal $\lambda_{\text{max}}^{\text{M}}$ are shown as black diamonds with error bars showing standard deviations. **(B)** Performance of the luminance system. *z* score (Equation 9) is the mean over 100 repetitions on differently seeded random mosaics. Key: cpd = cycles per degree.

The results are presented numerically in the upper row of Table 2 and visually in Figure 5. For the red-green system (Figure 5A), the optimal $\lambda_{max}^{\text{M}}=$ ~525 nm, with standard deviations of ~1 nm and a 95% confidence interval of <0.5 nm at all spatial frequencies. There is a small but significant change in $\lambda_{max}^{\text{M}}$ with spatial frequency [*p* = 8.9 × 10^−12^, α = 0.01, two-sample *t*-test between data for highest ($\lambda_{max}^{\text{M}}=$ 525.8 nm) and lowest ($\lambda_{max}^{\text{M}}=$ 524.84 nm) spatial frequencies]. The red-green system performs relatively better than the luminance system (Figure 5B) at all $\lambda_{max}^{\text{M}}$ positions in the range we tested. For both systems, discrimination performance increases as spatial frequency decreases. Means, standard deviations and confidence intervals are not given for the luminance system as the optimal $\lambda_{max}^{\text{M}}$ always occurs at the longest wavelength available. A note on the results of the luminance system: while it is typical to observe size-tuned responses in the luminance system, our results do not show this. Instead, the tuning curves for lower spatial frequencies have higher *z* scores. It has been observed that contrast sensitivity and the band-pass shape decrease as luminance levels decrease (Enroth-Cugell and Robson, 1966). As our goal here is to provide a comparison for the red-green system, we have used the same spectral images, and these contain fruit and leaf spectra are highly similar in intensity. Thus, the higher performance at lower spatial frequencies is a result of the low contrast of red-green gratings to the luminance system.

**Table 2:** The optimal $\lambda_{\text{max}}^{\text{M}}$ across spatial frequencies for the red-green system when spectral images are generated with (upper row) and without (bottom row) luminance variation.

**Spatial frequency**	**4 cpd**	**2 cpd**	**1 cpd**	**0.5 cpd**
Optimal $\lambda_{max}^{\text{M}}$ (lum. var.)	525.8 (0.92) [526.03, 525.57]	525.14 (0.95) [525.33, 524.95]	524.69 (0.92) [524.87, 524.51]	524.84 (0.60) [524.96, 524.72]
Optimal $\lambda_{max}^{\text{M}}$ (no lum. var.)	483.68 (14.15) [486.45, 480.91]	466.22 (16.58) [469.47, 462.97]	453.81 (7.11) [455.20, 452.42]	444.38 (4.65) [445.29, 443.27]

*The mean optimal $\lambda_{\text{max}}^{\text{M}}$ over 100 repetitions on differently seeded random mosaics are given, with the standard deviations provided in brackets. cpd, cycles per degree; lum. var., luminance variation. All results are in nanometres, standard deviations are given in (round brackets), upper and lower confidence intervals are given in [square brackets] for a confidence level of 95% (t = 1.96)*.

### 4.4. Luminance variation simulations

We aim to determine the effect that luminance variation has on the position of the optimal $\lambda_{max}^{\text{M}}$. luminance variation is a source of spatial noise—i.e., it causes different regions of the cone image to be active to different degrees even when stimulated by the same material. As such, it is a noise source that could not be included in previous works that did not model spatial dimensions. We applied the long-wavelength limit by fixing $\lambda_{max}^{\text{L}}=$ 562 nm, and used a broader but coarser search range for $\lambda_{max}^{\text{M}}$ of 440–560 nm as during testing we observed a shift to shorter wavelengths. As our aim with this test is only to identify qualitative shifts in the optimal $\lambda_{max}^{\text{M}}$ due to luminance variation, we reduce the sampling interval to 10 nm. To remove the luminance variation, the luminance coefficients are all set to 1 when creating the spectral images.

**Figure 6:**
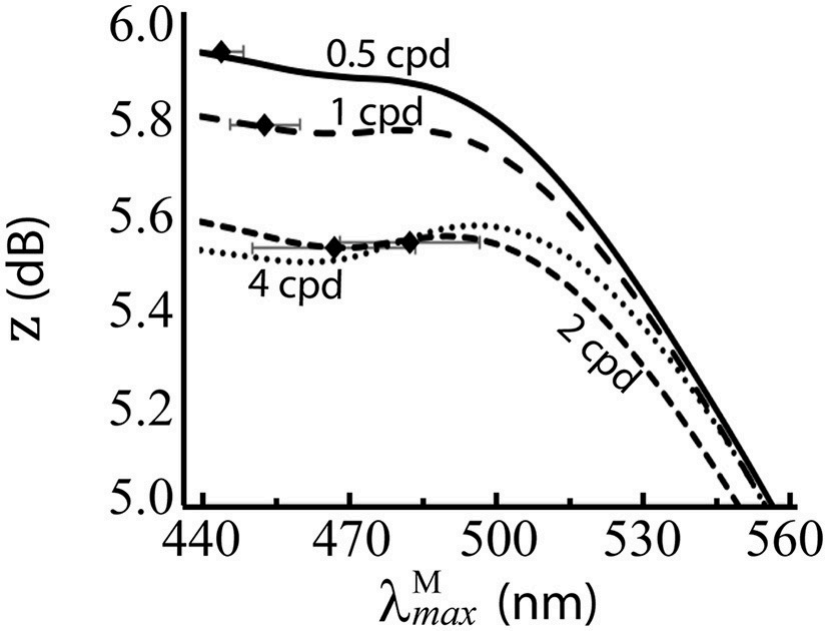
Performance of $\lambda_{\text{max}}^{\text{M}}$ for the red-green system at different spatial frequencies, when spectral images contain no luminance variation. *z* score (Equation 9) is the mean over 100 repetitions on differently seeded random mosaics. Mean optimal $\lambda_{\text{max}}^{\text{M}}$ are shown as black diamonds with error bars showing standard deviations. The four lines are for different spatial frequencies. Key: cpd = cycles per degree.

The results are presented numerically in the bottom row of Table 2 and visually in Figure 6. Without luminance variation, $\lambda_{max}^{\text{M}}$ in the range 440–500 nm lead to similar performance, but at longer wavelengths than ~500 nm, performance decreases with wavelength. There is a significant effect of spatial frequency, with the optimal $\lambda_{max}^{\text{M}}$ occurring at a longer wavelength for higher spatial frequencies (*p* = 5.2 × 10^−31^, α = 0.01, two-sample *t*-test between data for highest and lowest spatial frequencies: $\lambda_{max}^{\text{M}}=$ 483.68 nm and $\lambda_{max}^{\text{M}}=$ 444.38 nm, respectively).

## 5. Discussion

Trichromatic primate retinas encode chromatic and spatial information with a single 2-dimensional array of photoreceptors. Here we have shown that for the task of discriminating chromatic gratings formed of natural fruit and leaf spectra, and in the presence of natural luminance variation, the predicted optimal tuning of $\lambda_{max}^{\text{M}}=$ 525 nm is close to the naturally observed value of 535 nm. By comparison, models that consider chromatic responses independently of space predict a shorter wavelength $\lambda_{max}^{\text{M}}$, assuming fixed $\lambda_{max}^{\text{S}}$ and $\lambda_{max}^{\text{L}}$ (Osorio and Vorobyev, 1996; Regan et al., 1998; Sumner and Mollon, 2000a; Lewis and Zhaoping, 2006). This result assumes that $\lambda_{max}^{\text{L}}$ is fixed at its natural value of about 562 nm; in common with some (Osorio and Vorobyev, 1996; Lewis and Zhaoping, 2006) other models we find the value of $\lambda_{max}^{\text{L}}\approx$ 562 nm is suboptimal, implying that some factor other than spatio-chromatic discrimination (as modelled here) limits the evolutionary shift of the pigment to longer wavelengths: possibly the effects of dark-noise (Ala-Laurila et al., 2004). The optimal value of $\lambda_{max}^{\text{M}}$ is affected by the luminance variation, which causes $\lambda_{max}^{\text{M}}$ to occur closer to $\lambda_{max}^{\text{L}}$ than it would if luminance were perfectly uniform across the scene. We also confirmed that the particular reflectance spectra of fruit and leaves are significant in setting this optimum, as a very different optimum (with much less spectral overlap) is predicted when the more diverse spectra of Munsell chips are used to build the images.

This more accurate prediction of the M cone spectral sensitivity has an important implication for the role of the luminance system in constraining the separation of $\lambda_{max}^{\text{M}}$ from $\lambda_{max}^{\text{L}}$. Previously, it was believed that the requirement of the luminance system for a more correlated signal provided a selective pressure for more overlapping M and L cone spectral sensitivities (Osorio et al., 1998; Vorobyev, 2004) that was strong enough to drive $\lambda_{max}^{\text{M}}$ from its computationally predicted optimal position to its naturally observed position—a shift of around 20 nm. However, our spatio-chromatic analysis results in the discrepancy between the predicted and observed $\lambda_{max}^{\text{M}}$ being reduced to around 10 nm. This suggests that the purported selective pressure from the luminance system may be weak, in which case, the luminance system may not be greatly impaired by the decorrelation of M and L cone spectral sensitivities. This is in agreement with the recent empirical finding that high-acuity luminance vision is not worse in trichromatic female marmosets than in their dichromatic male counterparts, despite the fact that the parvocellular channel of the trichromats also carries the red-green signal (Martin et al., 2011). However, a number of studies on human dichromats have reported advantages over trichromats in certain laboratory conditions (Dain and King-Smith, 1981; Schwartz, 1994; Jägle et al., 2006; Sharpe et al., 2006; Melin et al., 2007, 2010; Caine et al., 2010; Janáky et al., 2014), and further research will be required before this question is answered definitively.

We predict the M cone spectral tuning by modeling the detectability of chromatic gratings; a task which can be compared to that of finding fruit amongst leaves in a complex natural environment (cf. Párraga et al., 1998, 2002). In addition to a red-green signal, the model generates a luminance signal. Comparing the exhaustive search results for the red-green and luminance systems, we observe that red-green vision leads to higher discrimination performance at all $\lambda_{max}^{\text{M}}$ in the range tested. This supports the theory that M and L cone spectral sensitivities are well-adapted for fruit foraging (Osorio and Vorobyev, 1996), but says nothing about how this task compares with other tasks implicated in the spectral tuning of primate M and L cones, such as young-leaf foraging (Sumner and Mollon, 2000a, 2003) or social signalling (Changizi et al., 2006; Hiramatsu et al., 2017), or whether the facilitation of fruit foraging was the underlying cause of the evolution of primate trichromacy. The only other scenario upon which the model was tested was the database of highly varied spectra which was used as a control to ensure that our model was not biased toward predicting highly correlated M and L cone spectral sensitivities (Figure 4A).

There is a small but significant change in $\lambda_{max}^{\text{M}}$ with spatial frequency between the lowest and highest spatial frequencies tested. Despite the high significance of this result, the small difference in the optimal $\lambda_{max}^{\text{M}}$ between the highest and lowest spatial frequencies (1.1 nm) and the fact that other neighbouring $\lambda_{max}^{\text{M}}$ have only slightly lower performance—as shown by the performance curves in Figure 5A— suggest this effect may be negligible.

The effect the luminance variation which occurs in natural scenes has upon spectral sensitivities was investigated via a comparison of the optimal $\lambda_{max}^{\text{M}}$ when natural luminance variation is (Figure 5A) and is not (Figure 6) included in the spectral images. Without luminance variation, our model predicts the optimal $\lambda_{max}^{\text{M}}$ in the range 440–500 nm. Such a distributed configuration of the peak sensitivities is typical of colour vision systems, as seen in many animals with colour vision (Osorio and Vorobyev, 2008) and demonstrated here using Munsell spectra, which cover the full colour gamut of for trichromatic primates. In the luminance variation simulations, the only source comes from the sampling of the scene by different cone types at different locations. At the higher spatial frequencies, there are fewer cones per cycle, so the noise introduced by the random cone mosaic has a greater impact, and leads to the optimum $\lambda_{max}^{\text{M}}$ occurring around 500 nm, whereas at the lower spatial frequencies, it tends to the shortest wavelength it can occupy (440 nm). Because this spatial-frequency-dependent effect on the optimal peak sensitivity is largely lost when luminance variation *is* included in the spectral images, this suggests that the tuning of $\lambda_{max}^{\text{M}}=$ 525 nm in the Optimal $\lambda_{max}^{\text{M}}$ simulations is heavily influenced by luminance variation. This conclusion is similar to that of (Sumner and Mollon, 2000a), who found that the high correlation of M and L spectral sensitivities minimised the variance in chromaticities of the background leaves.

## Author contributions

TM devised the concept, created the model, and wrote the manuscript; DO gave substantial contributions to the concept, interpretation of data, and drafting of the manuscript; AC gave substantial contributions to the concept, and drafting of the manuscript; LC gave substantial contributions to the concept, interpretation of data, and drafting of the manuscript.

### Conflict of interest statement

The authors declare that the research was conducted in the absence of any commercial or financial relationships that could be construed as a potential conflict of interest.
